# Antiseptics and the War

**Published:** 1915-08-21

**Authors:** 


### ANTISEPTICS AND THE WAR.

The rival merits of antiseptic surgery and of aseptic surgery have provoked many discussions, and not a few authorities were inclined to the view that the latter could, at the expense of its rival, look forward to a confident future. But with the war the position greatly changed. For the strict conditions of the operating-theatre and the clean wound were substituted injuries inflicted in circumstances in which sepsis was an almost inevitable event. Antiseptic practice seemed to come into its own again, and the value of the thorough application of carbolic acid received the endorsement of an official circular. It is true that there were challenging voices, but the offer to fight was treated with neglect. The wheel had come full circle, and the antiseptic surgeon once more reigned supreme in the military department.

But ere long there came a disturbing voice—a voice which threw doubt in a region where conviction had previously seemed secure. That antiseptics could "hold off" microbes from the clean wounds made in surgical operations and could thus prevent infection of these wounds, was allowed. But whether these agents could destroy microbes in wounds already septic was, it was urged, another question, and it is with wounds of this order that the military surgeon is concerned. Further and new infections might be prevented, but would the one already effected be destroyed? To say that the antiseptic agent would at least overcome many or most of the microbes, and would thus certainly effect a partial sterilisation, is not enough, for in view of the rapid multiplication of microbes the number present in the wound in the course of a day or two would be sufficient to leave the position essentially unchanged. Hence it became a question whether, while allowing the *prophylactic* value of antiseptics as a means of preventing wound infection, their use as *curative* agents in the case of wounds already infected was not largely a mistake. In opposition to such use we were introduced to "physiological" methods of treatment, to the natural anti-bacterial agencies of the body, and to the virtues of the phagocytes and of anti-bacterial lymph. All unconsciously, the surgeon, we were told, was bringing these forces into operation by his incisions and free drainage and hot fomentations. But it is possible—such is the claim—still more efficaciously to arouse the natural tissue capacities by the application of certain saline solutions; these, it is said, will increase the volume of the anti-bacterial lymph and so secure a sustained and effective action against the invading microbes lurking in the recesses of the wound or lodged within its walls. No one can fail to admire the ingenuity of the reasoning and the dexterity of the experiments on which these claims are based. Yet it may fairly be asked whether the present circumstances are suitable for the renunciation of methods which can certainly boast much practical value, and to the substitution for them of a system which rests mainly on laboratory investigations. That sooner or later this system must be subjected to an extended trial may cheerfully be allowed, but it can hardly be urged upon practitioners of whom many have neither studied its bases nor been trained in its practice. The matter is one for investigation, but it has not yet reached the stage which would justify its substitution for the approved value of antiseptic treatment.

In the circumstances here related there is a special interest attached to the researches which have established the possibility of obtaining the virtues of hypochlorous acid in a form suitable for wound treatment. The "new antiseptic" can be applied as a dry powder, and may thus be used even when water for solution is not at hand. While its anti-bacterial action is pronounced it has no irritating or prejudicial action on the tissues, and it promotes the flow of lymph, which Sir Almroth Wright presents as the essential aim of his "physiological" treatment.

